# Design, synthesis and application of two-dimensional metal tellurides as high-performance electrode materials

**DOI:** 10.3389/fchem.2022.1023003

**Published:** 2022-09-26

**Authors:** Meng Guo, Shaonan Gu, Shuzheng Xu, Jiani Lu, Yinan Wang, Guowei Zhou

**Affiliations:** Key Laboratory of Fine Chemicals in Universities of Shandong, Jinan Engineering Laboratory for Multi-scale Functional Materials, School of Chemistry and Chemical Engineering, Qilu University of Technology (Shandong Academy of Sciences), Jinan, China

**Keywords:** 2D metal tellurides, synthesis, electrocatalysis, batteries, electrodes

## Abstract

Multifunctional electrode materials with inherent conductivity have attracted extensive attention in recent years. Two-dimensional (2D) metal telluride nanomaterials are more promising owing to their strong metallic properties and unique physical/chemical merits. In this review, recent advancements in the preparation of 2D metal tellurides and their application in electrode materials are presented. First, the most available preparation methods, such as hydro/solvent thermal, chemical vapor deposition, and electrodeposition, are summarized. Then, the unique performance of metal telluride electrodes in capacitors, anode materials of Li/Na ion batteries, electrocatalysis, and lithium-sulfur batteries are discussed. Finally, significant challenges and opportunities in the preparation and application of 2D metal tellurides are proposed.

## 1 Introduction

Increasing concerns about scarce resources and global climate issues have promoted the pursuit of clean and renewable energy for all humanity (Goodenough, 2014; Zeng et al., 2019). Many initiatives have attempted to power industrial civilization with renewable energy while ensuring the economic viability of related technologies, such as supercapacitors (SCs), alkali-ion batteries, lithium-sulfur (Li-S) batteries, etc., (Jaiswal, 2017; Zhang and Guo, 2020; Elmorshedy et al., 2021). Since then, electrode materials have emerged as a popular research topic in recent years. Many studies have attempted to improve the electrochemical characteristics of electrode materials by changing their composition, nano/microstructures, electronic properties, and so on (Zhang et al., 2019a; Zheng et al., 2021). However, as electrodes, one of the most important considerations is the inherent conductivity of the material.

Tellurium (Te), a sulfur element, has a higher conductivity compared with sulfur and selenium. It also has strong metallic characteristics, allowing telluride materials to admit more electrolyte ions and increase diffusion kinetics, enhancing energy storage reaction and offering high rate capability of energy storage devices (Kshetri et al., 2021). Owing to the unusual electrical structures and various two-dimensional (2D) crystals of 2D metal tellurides, these materials have recently received widespread attention as an essential component of metal chalcogenides (Li et al., 2016; Apte et al., 2018; Kononov et al., 2020; Liu et al., 2020). For instance, VTe_2_ has excellent electrocatalytic activity for hydrogen evolution reactions and is regarded as a high-performance electrode material (Shi et al., 2021). In addition, NiTe, which has better conductivity and faster electron transfer capability compared with semiconductors, can maintain a specific capacity of approximately 307 mAh g^−1^ at a high rate of 500 mA g^−1^ as the anode of rechargeable aluminum ion batteries (Yu et al., 2020). Thus, the study of cathode materials for aluminum ion batteries and their use as battery anode materials [e.g., FeTe_2_ (Park and Kang, 2020) and CoTe_2_ (Yang et al., 2020)], electrocatalytic materials [e.g., Ni_3_Te_2_ (De Silva et al., 2018) and MoTe_2_ (Zhou et al., 2017a)], and SC materials [e.g., NiTe (Chen et al., 2019) and CoTe_2_ (Manikandan et al., 2020)] is of great importance.

**FIGURE 1:**
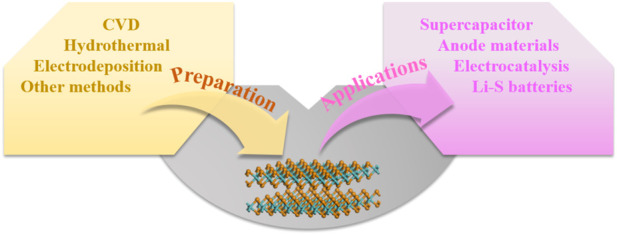
Overview framework of 2D metal telluride atomic crystals.

In recent years, tellurides have become widely used in electrochemistry owing to their 2D layered structure and unique properties (Wang et al., 2019; Li et al., 2020a; Myung et al., 2020; Zhang et al., 2021). On this basis, we provide a comprehensive overview of tellurides in this study, as shown in Figure 1. We first focus on the preparation methods of telluride electrode materials, including chemical vapor deposition (CVD) (Wood et al., 2014; Zhou et al., 2017b; Tang et al., 2019; Hao et al., 2021; Zhang et al., 2021; Zhou et al., 2021), hydrothermal method (Wang et al., 2012; Hou et al., 2013; Oh et al., 2020; Qi et al., 2021), and electrodeposition (Morris and Vanderveen, 1993; Yu et al., 2018), and their electrochemical properties and applications. Subsequently, conceivable perspectives on the challenges and opportunities of 2D telluride electrode materials are proposed to provide insights into future research.

## 2 Preparation

2D metal tellurides have recently become popular in energy devices such as SCs, photocatalysts, and electrode materials. Thus far, several preparation methods have been developed to synthesize 2D metal telluride nanomaterials, as summarized in Table 1.

**TABLE 1:** Synthesis methods and application of metal tellurides.

Tellurides	Applications	Preparation method	Morphology	Temp (◦C)	Time (h)	Ref
Bi_2_Te_3_	-	Hydrothermal	Nanotubes	180	48	Wang et al. (2010)
	Thermoelectric application	Spark plasma sintering	Nanosheet	260	-	Kim et al. (2021)
BiSbTe_3_	LIBs	Solvothermal	-	180	24	Zhu et al. (2020)
WTe_2_	Superconductors	-	Film	350	-	Asaba et al. (2018)
CoTe_2_	-	Solvothermal	Nanotubes	200	24	Li et al. (2009)
	Electrocatalyst	Hydrothermal	Nanoparticles	180	16	Lu et al. (2015)
	Li-S batteries	Hydrothermal	-	200	24	Song et al. (2021)
MoTe_2_	Li-S batteries	Hydrothermal	-	200	36	Yu et al. (2021)
	Photocatalysts	Hydrothermal	-	200	48	Li et al. (2020b)
VTe_2_	Li-S batteries	CVD	-	∼650	-	Wang et al. (2019)
Sb_2_Te_3_	LIBs	Mixing	-	-	-	Grishanov et al. (2018a)
	-	Microwave-assisted Solvothermal	Various morphologies	-	-	Dong et al. (2011)
	LIBs	Ball milling	Micro-particles	-	12	Wei et al. (2020)
Ge_2_Sb_2_T_5_	LIBs	Ball milling	-	-	40	Wei et al. (2019)
NiTe	Supercapacitor Electrode material	Solvothermal	Nanoplates	200	20	Chen et al. (2019)
		Hydrothermal	Network	180	18	Deshagani et al. (2020)
SnTe	LIBs	Hydrogen peroxide	-	-	-	Grishanov et al. (2018b)
Cu_2_Te	-	Electrodeposition	-	-	-	Ishizaki et al. (2003)
Cu_(2-x)_Te	Chemotherapy	Bio-synthesis	Nanocubes	160	0.75	Poulose et al. (2016)
ZnTe	Bio-imaging and bio-labeling	Bio-synthesis	Nanoparticles	-	-	Dunpall et al. (2014)
MnTe	Optoelectronic devices	CVD	Nanosheet	580	-	Li et al. (2020a)
In_2_Te_3_	Gas sensing and hydrogen storage	Solvothermal	Nanotubes	180	48	Zhou et al. (2014)

### 2.1 Hydro/solvothermal methods

The most prevalent techniques for synthesizing nanomaterials are the hydrothermal and solvothermal procedures. In contrast to alternative procedures for synthesizing nanostructured materials, the hydrothermal approach offers the benefits of low synthesis temperature and small grain size. This approach may produce a wide range of morphologies, including nanorods (Yu et al., 2020; Jayababu et al., 2021), nanosheets (Lu et al., 2015; Li et al., 2020b), nanowires (Yang et al., 2009; Wan et al., 2011), and nanotubes (Wang et al., 2010). Moreover, in contrast to other synthesis methods, the hydrothermal approach has an excessively high oxygen affinity for positively charged metal and negatively charged Te ions, resulting in the formation of oxides. Reducing agents, such as hydrazine and sodium borohydride, are necessary to avoid oxide impurities in tellurides.

**FIGURE 2:**
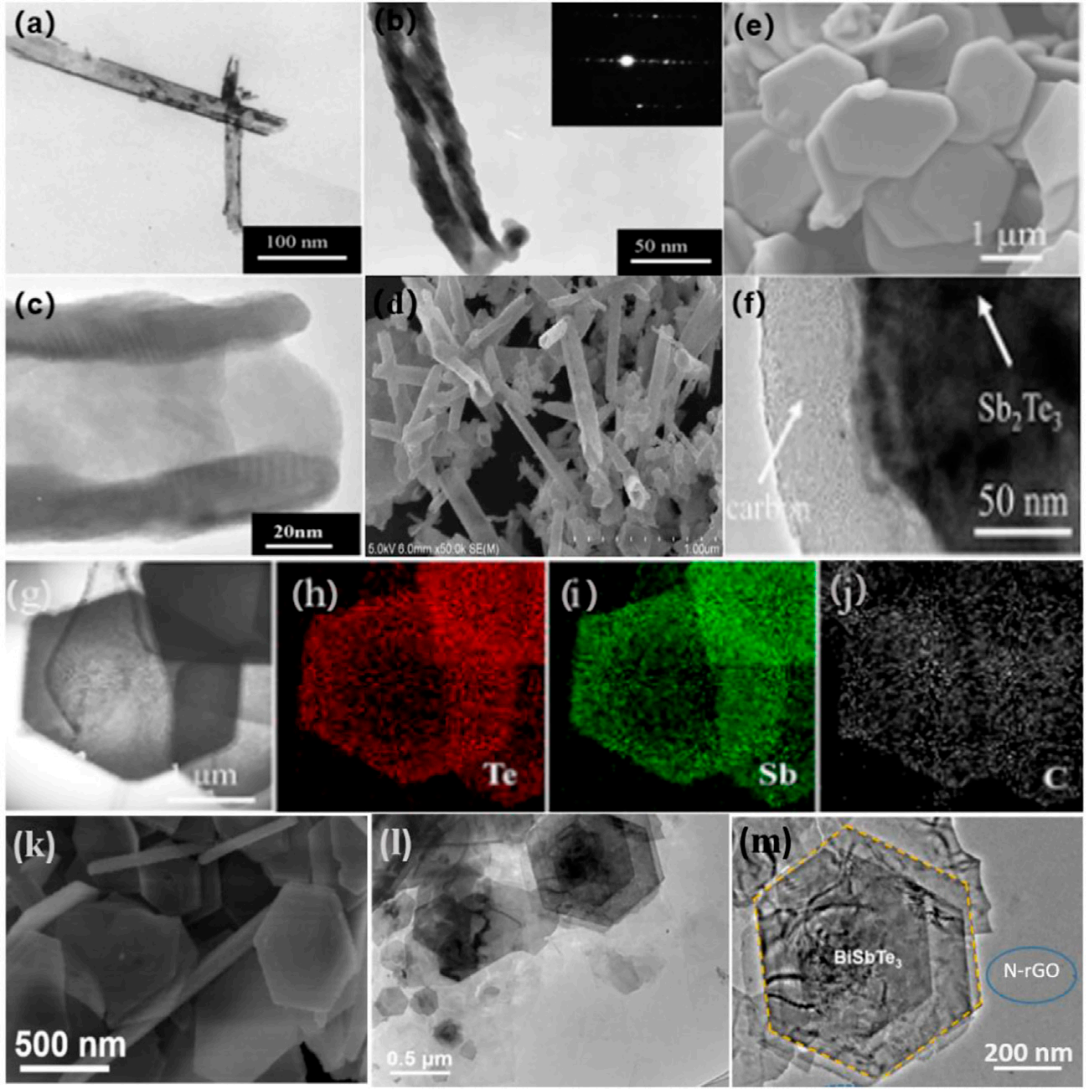
**(A–D)** TEM, SAED pattern, and SEM images of the Bi_2_Te_3_ (Wang et al., 2010). Copyright 2010, Elsevier. **(E–J)** morphologies and Te, Sb, C elemental mapping of the as-prepared Sb_2_Te_3_@C sample (Zhang et al., 2019b). Copyright 2019, American Chemical Society. **(K)** SEM of BiSbTe_3_ nanosheets. **(L)** TEM and **(M)** HRTEM images of BiSbTe_3_/N-rGO (Zhu et al., 2020). Copyright 2020, Elsevier.

Wang et al. adopted the hydrothermal technique to synthesize Bi_2_Te_3_ nanotubes with diameters of 100 nm and lengths of 500–1,000 nm by using sodium borohydride as the reducing agent and EDTA as the surfactant (Figure 2A–D) (Wang et al., 2010). Zhang et al. created carbon-encapsulated porous Sb_2_Te_3_ nanoplates with porous architectures *via* the hydrothermal and carbonization processes, with carbon shell thicknesses ranging from 50 to 80 nm (Figures 2E–J) (Zhang et al., 2019b). Their approach can successfully produce nanomaterials, such as Ag_2_Te (Zhang et al., 2006), Bi_2_Te_3_ (Wang et al., 2010), CdTe (Gong et al., 2011), Cu_2_Te (Zhang et al., 2006), Cu_2-x_Te (Jamwal et al., 2016a), HgTe (Salavati-Niasari et al., 2010), NiTe (Zhang et al., 2002), and PbTe (Jamwal et al., 2016b).

Similar advantages can be attained in solvothermal reactions, such as when one or more precursors are dissolved in a non-aqueous solvent. The BiSbTe_3_ nanosheets were prepared under solvent-heated conditions by using ethylene glycol as the solvent (Figures 2K–M) (Zhu et al., 2020). The unique nano-plate structure of BiSbTe_3_ increases the exposure of the electrolyte, leading to high utilization of the composite electrode material during cycling. In addition, the solvothermal method is also commonly used to prepare other metal tellurides, such as Bi_2_Te_3_ (Liu et al., 2017a), Cu_*x*_Te (Liu et al., 2017a), PbTe (Liu et al., 2017a), Ag_2_Te (Liu et al., 2017a), and Sb_2_Te_3_ (Yan et al., 2016).

### 2.2 Chemical vapor deposition methods

CVD is generally used to create thin film materials. This approach is based on the principle of utilizing gaseous precursor reactants to produce thin films on a substrate by breaking down specific components of the gaseous precursor *via* atomic and intermolecular chemical interactions (Zhou et al., 2015; Naylor et al., 2016; Zhou et al., 2017b; Yoo et al., 2017). The form and properties of the 2D metal tellurides are influenced by the substrates, precursors, and temperature, among other factors (Zhou et al., 2016; Zhang et al., 2019c; Huang et al., 2019; Kim et al., 2020). CVD method is the most widely used method for the synthesis of tellurides, which has good scalability and can be controlled to prepare large-area films or two-dimensional crystals.

**FIGURE 3:**
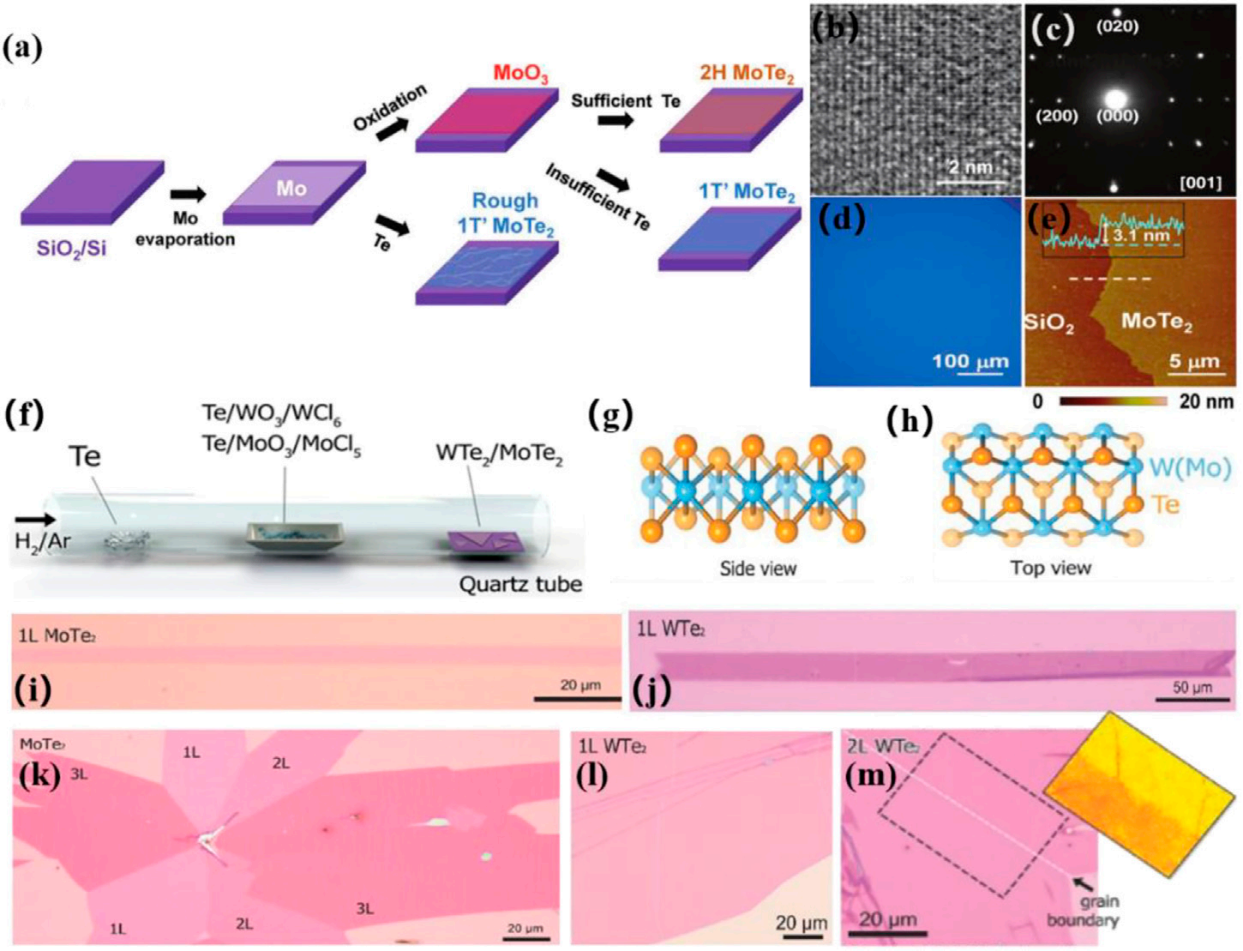
**(A)** Schematic illustration of CVD method to deposit 1T′ and 2H MoTe_2_ films. **(B)** high-resolution TEM image, and **(C)** SAED pattern of 1T′- MoTe_2_ film grown from MoO_3_; **(D)** Typical optical and **(E)** AFM image of a 1T′- MoTe_2_ film (Zhou et al., 2016). Copyright 2016, Wiley-VCH. **(F)** Schematic illustration of CVD method to grow WTe_2_ and MoTe_2_ atomic layers. **(G,H)** Crystalline structures of 1T′-W(Mo)Te_2_. **(I,J)** Optical images of MoTe_2_ and WTe_2_ monolayer. **(K)** Optical image of a MoTe_2_ flake containing 1, 2, and 3 L MoTe_2_. **(L,M)** Optical images of a large scale monolayer and bilayer WTe_2_ films (Zhou et al., 2017b). Copyright 2017, Wiley-VCH.

Recently, MoTe_2_ has received widespread attention owing to its distinct semiconducting and semi-metallic characteristics. Kong et al. investigated a CVD technique for fabricating uniform high-crystalline 2H and 1T'-MoTe_2_ films (Naylor et al., 2016). Various products were created by varying the precursor, carrier gas, and temperature. Under the same conditions, they observed that MoO_3_ precursors converted more easily into 2H-MoTe_2_, whereas MoO and MoO_x_ (x < 3) precursors converted more effectively into 1T'-MoTe_2_. A year later, Kong et al. improved the preparation process and successfully synthesized large-size homogeneous 1T'-MoTe_2_ (Zhou et al., 2016). They further determined the significant effect of the molybdenum precursor on the formation of 1T'-MoTe_2_ (Figure 3A–E). 1T'-MoTe_2_ was reliably created when MoO_3_ was utilized as a precursor. Furthermore, the amount of Te used in the synthesis of 1T'-MoTe_2_ had a substantial impact. If the Te supply is sufficient, then 2H-MoTe_2_ would be produced; otherwise 1T'-MoTe_2_ would be produced (Zhou et al., 2016). The established CVD technique also allowed for the large-scale direct synthesis of WTe_2_ and MoTe_2_ multilayers and monolayers (Zhou et al., 2017b). The thickness of the WTe_2_ and MoTe_2_ atomic layers was adjusted using growth time. (Figure 3F–M).

In addition to 2D WTe_2_ and MoTe_2_, many other 2D transition metal tellurides can be grown *via* CVD methods. Li et al. reported a method that can precisely control 2H-MoTe defects grown by a large-scale phase-change-assisted CVD process using selective etching of I_3_^−^ solutions (Zhou et al., 2021). Liu et al. reported a facile CVD method to synthesize Mo_x_W_1-x_Te_2_ with controlled thickness and chemical composition ratios to investigate its design of material devices from a topological quantum state perspective (Chubilleau et al., 2011). Li et al. reported a strategy using mixed molten salts for enhancing the CVD growth of 2D WTe_2_ crystals with large grain size and yield, acting as a synergist (Jayababu et al., 2021).

### 2.3 Electrochemical deposition method

Electrochemical deposition (ECD) is another effective method for obtaining metal tellurides. ECD has the outstanding advantage of easily controlling the morphologies of metal tellurides by using removable templates. Compared with other methods, the ECD method is simple, not limited by grain size and shape, and the prepared crystalline materials have unique properties. Applied electrical potential and deposition rate are two critical parameters of smooth ECD.

**FIGURE 4:**
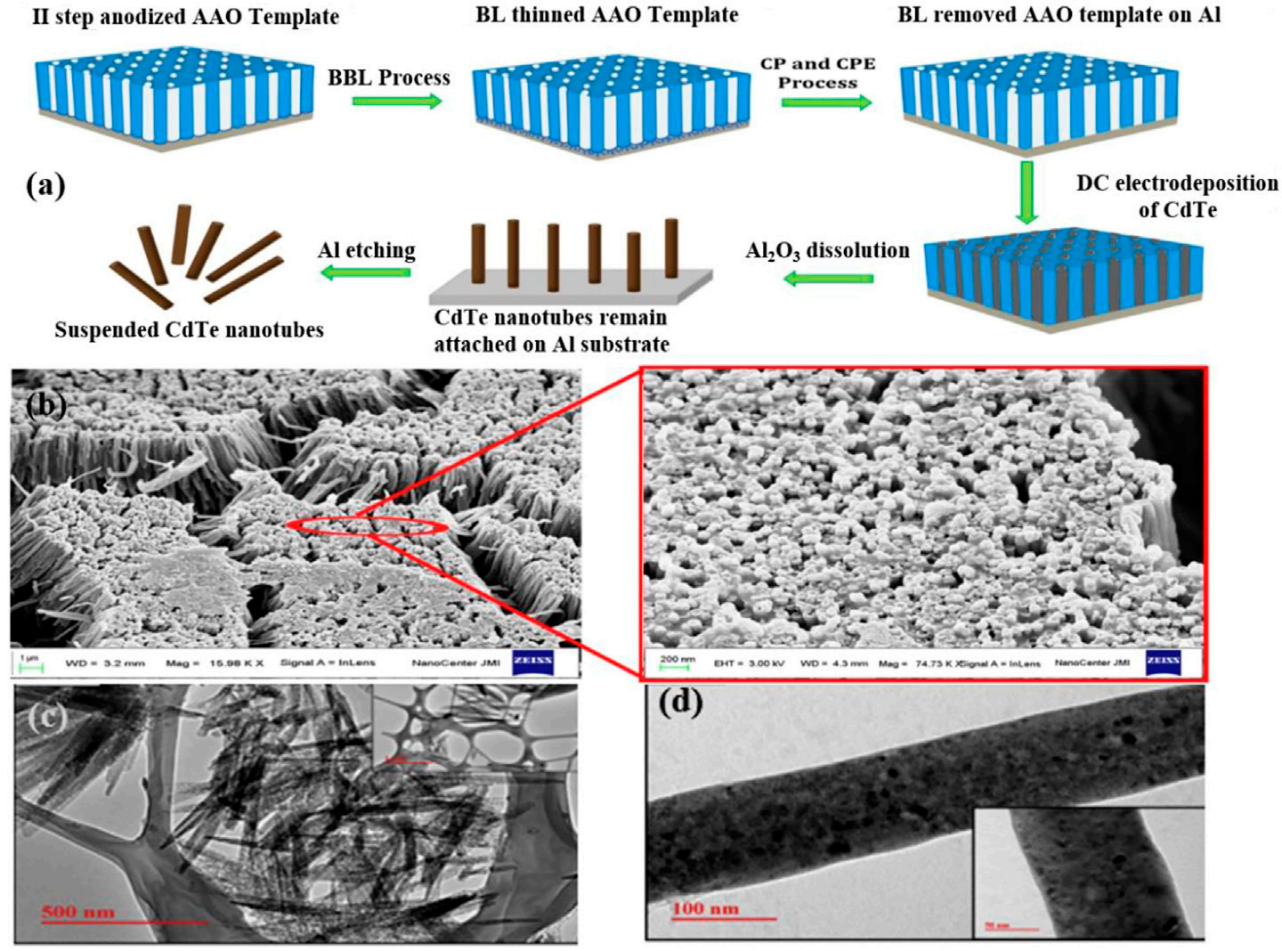
**(A)** Schematic illustration of the steps involved to remove barrier layer from AAO template keeping base Al intact for electrodeposition of CdTe nanotubes; and **(B)** FE-SEM micrograph of CdTe nanotubes deposited in BL removed AAO template. **(C)** TEM images illustrating CdTe hollow nanotubes; and **(D)** solid nanorod obtained using sulfate electrolyte bath (Wu et al., 2020). Copyright 2019, Elsevier.

Islam et al. suggested an ECD technique for handling bespoke Al_2_O_3_ (AAO) stencils that neither needed extensive hole branching nor would damage the aluminum substrate(Figure 4) (Wu et al., 2020). CdTe nanotubes have a high aspect ratio of one-dimensional nanostructures compared to other nanostructures and have amplified optical waveguide properties. Therefore, they are designed as visible light responsive photocatalysts. Figure 4A shows that CdTe was electrochemically deposited onto tailored AAO stencils, which has been sheared by utilizing the full AAO to allow for the acquisition of through-hole and self-supporting features on the Al substrate. The CdTe nanotubes developed in the sulfate bath after the barrier layer has been completely removed, as shown in Figure 4B. The image within the red border is a magnified view of the designated section in the red circle, revealing the hollow ends of the vertically aligned nanotubes. This feature implies the significant advantage of the material to provide electrical contact during cathodic deposition because the aluminum base remains intact even after the barrier layer is completely removed. Meanwhile, broaching barrier layer (BBL) was polarized in dilute H_2_SO_4_, compared to neutral KCl solution and immersion in H_3_PO_4_ solution, resulting in a totally etched barrier layer that was innocuous to the substrate aluminum.

**FIGURE 5:**
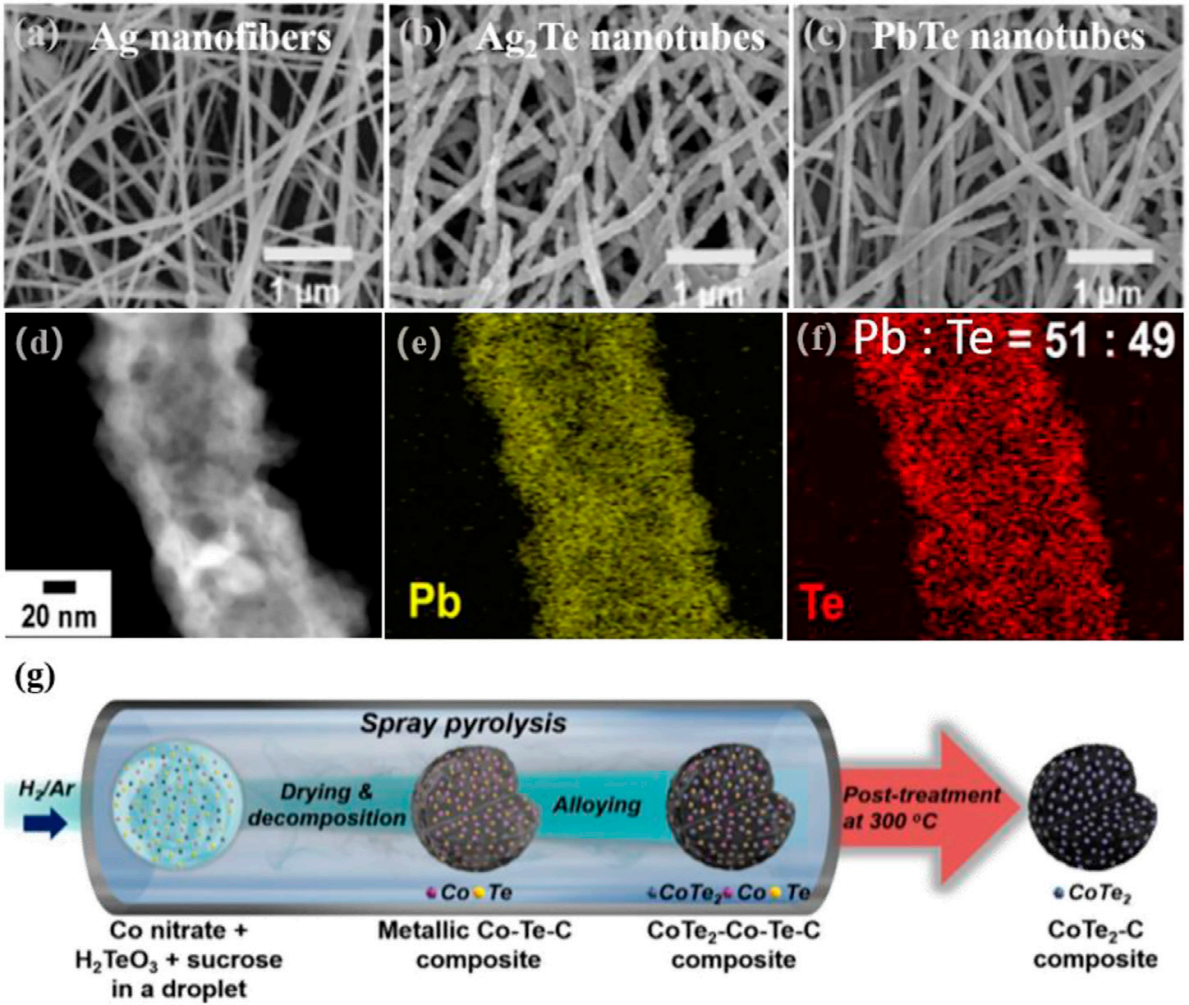
SEM images of **(A)** Ag nanofibers; **(B)** Ag_2_Te nanotubes and **(C)** PbTe nanotubes; **(D–F)** EDS mapping (Deshagani et al., 2020). Copyright 2018, Elsevier. **(G)** Schematic illustration of the synthetic process of CoTe_2_-C composite (Yang et al., 2020). Copyright 2020, Elsevier.

Choa et al. converted Ag_2_Te nanotubes to PbTe nanotubes by changing the silver-to-lead atomic ratio through the combined processes of electrostatic spinning, ECD, and cation exchange (Figures 5A–F) (Deshagani et al., 2020). Silver atoms were diffused into the Te layer and transformed into Ag_2_Te nanofibers by ECD using silver nanofibers synthesized by electrostatic spinning as the starting material. Then, the crystalline transition of Ag_x_Te_y_ to PbTe nanocomposites was controlled by the cation exchange from Ag^+^ cations to Pb^2+^ cations.

In addition to the abovementioned three methods, many other chemical routes can be used to extensively investigate the synthesis of metal telluride nanomaterials. Examples are microwave synthesis (Ye et al., 2019), spray pyrolysis (Chen et al., 2019), biosynthesis (Poulose et al., 2016), and laser ablation techniques (Jayababu and Kim, 2021). A good method allows for the accurate control of certain features, including spatial structure and distribution, which significantly impact the performance of electrode materials. Kang et al. investigated anode materials for potassium ion batteries using spray pyrolysis to make cobalt telluride-C (CoTe_2_-C) composite microspheres, as shown in Figure 5G, (Yang et al., 2020). As Te needed to be directly embedded into the composite microspheres, a simple one-step post-treatment technique was used to prepare CoTe_2_-C composite microspheres. Their approach could be explained by Ostwald maturation induced by the formation of the CoTe_2_ crystals.

## 3 Applications as electrodes

### 3.1 Supercapacitor

**FIGURE 6:**
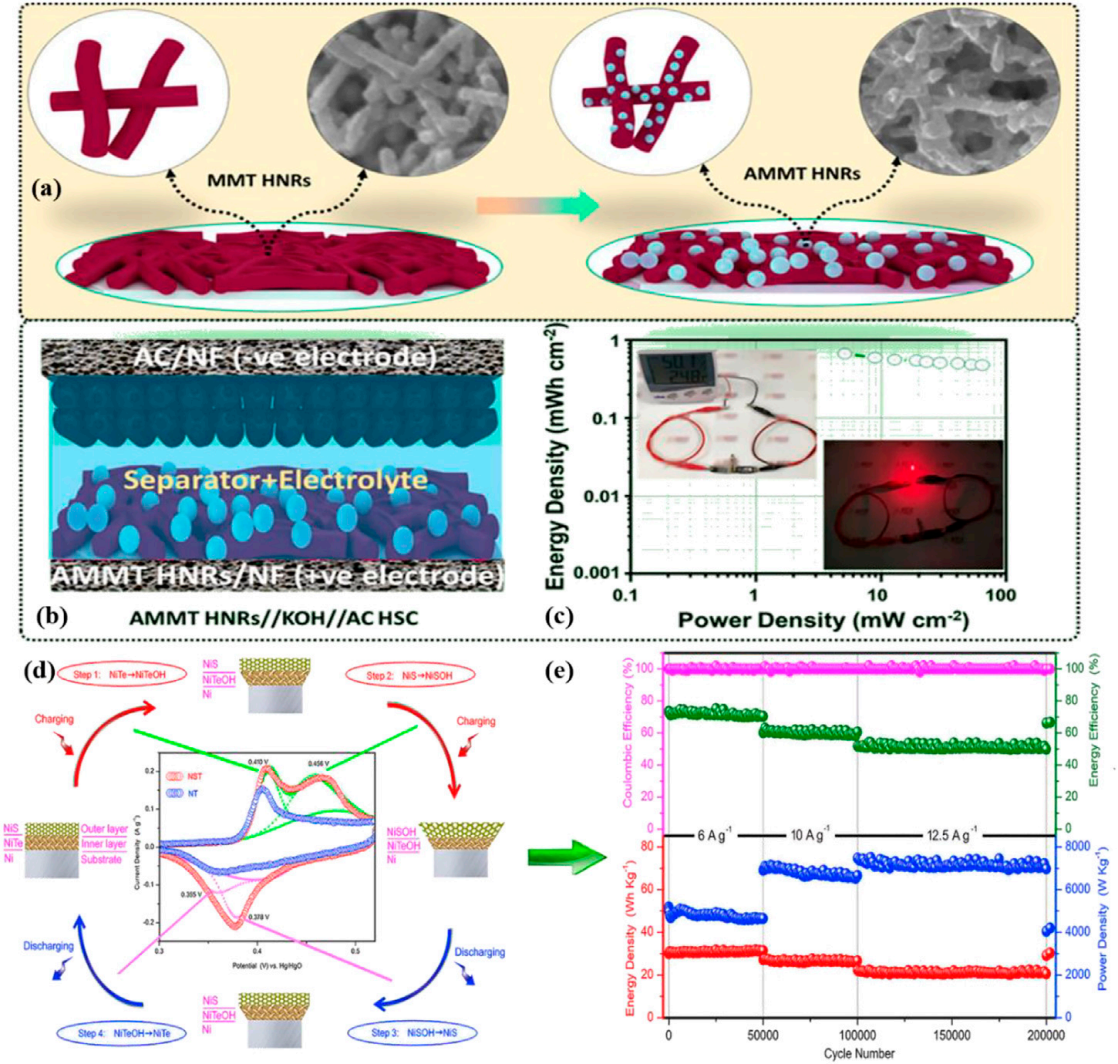
**(A)** Schematic of the MMT HNRs/NF and AMMT HNRs/NF electrodes; **(B)** Schematic of the hybrid supercapacitors; **(C)** Energy and power density of the hybrid supercapacitors and real-time suitability tests as a digital multi-sensor power supply (Jayababu et al., 2021). Copyright 2021, American Chemical Society. **(D)** Step by step illustration of NT and NST electrodes’ electrochemical processes. **(E)** Electrochemical performance of NiS/NiTe/Ni//AC asymmetric supercapacitor electrodes up to over 200 000 cycles at different current densities (Chen et al., 2019). Copyright 2019, Elsevier.

Telluride has a substantially greater electrical conductivity and is projected to perform better electrochemically compared with other materials, leading to the widespread research and advancement of metal tellurides built into diverse nanostructures for supercapacitor (SC) applications (Liu et al., 2017b; Rathore et al., 2022). Kim et al. created silver-decorated NiFe alloy telluride nanorods (AMMT HNRs) on nickel foam (NF) (Figure 6A) (Jayababu et al., 2021). The robust electroactivity of the NiFe alloy, the high conductivity of Te and Ag, and the porous layered structure of the telluride all contributed to the AMMT HNRs/NF electrode’s outstanding electrochemical performance. The electrode exhibited a stability of 80.4% over 3,000 cycles. After employing AMMT HNRs/NF and carbon-coated NF as positive and negative electrodes, respectively, and cellulose membranes as separators, high areal energy and power densities were reported in hybrid supercapacitors. (Figure 6B).

NiTe, as an SC electrode material, has also attracted the interest of scientists (Park et al., 2018; Song et al., 2021; Yu et al., 2021). The NiTe achieved outstanding electrochemical performance as a coexisting pseudo capacitive material for NiS reported by Wu et al. (Chen et al., 2019). As shown in Figure 6D, four electrochemical reactions occurred on the NiS/NiTe/Ni (NST) electrode. During charging, the volume increased from the inner layer to the outer layer; during discharge, the volume was reduced from the outer layer to the inner layer. The NST was the positive electrode of the asymmetric SC, whereas the active carbon (AC) was the negative electrode. The high capacitance retention and ultra-long cycle life (200000 cycles, Figure 6E) demonstrated the important role of the synergistic structure to structural stability. Additionally, CoTe_2_ (Manikandan et al., 2020), CuCoTe (Fu and Lee, 2019), VTe_2_ (Ahmad et al., 2021), and MoTe_2_ (Jin et al., 2018) have been widely used as electrode materials for high-performance capacitors.

### 3.2 Anode materials

**FIGURE 7:**
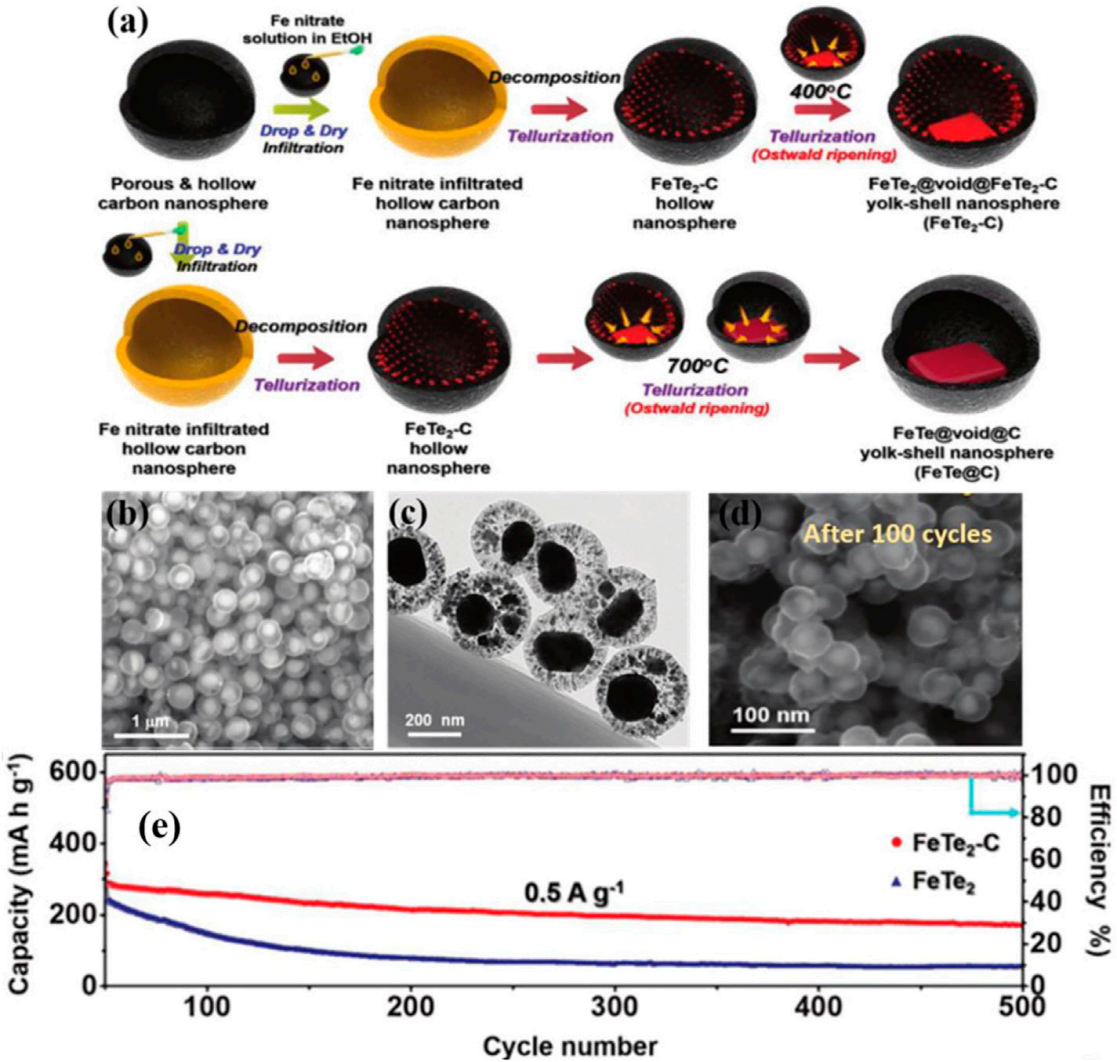
**(A)** Formation illustration of the hollow carbon nanosphere within FeTe_2_-C nanospheres: **(B–D)** SEM and TEM images of initial FeTe_2_-C and FeTe_2_-C after cycled. **(E)** long-term cycling performances of FeTe_2_ and FeTe_2_-C (Park and Kang, 2020). Copyright 2020, Wiley-VCH.

Metal tellurides have emerged as the most feasible alternative for cutting-edge ion battery anode materials because to their layered crystal structure, high intrinsic conductivity, and high trap density (Zhu et al., 2020; Guo et al., 2021; Hassan et al., 2021). Kang et al. used a structurally distinct FeTe_2_ and carbon nanocomposite as anode material for potassium ion batteries (Figure 7) (Park and Kang, 2020). The hollow carbon nanospheres that housed the iron telluride nanocrystals (FeTe_2_-C) offered enough space to accommodate for the nanocrystals’ enormous volume variations during charging and discharging. During cycling, nanocrystal extrusion within the solid hollow carbon nanospheres was controlled, and no FeTe_2_ from the electrode were lost, showing strong structural integrity (Park and Kang, 2020). Kang et al. also investigated the reaction mechanism of the CoTe_2_-C composite microsphere as an anode material for potassium ion batteries and the related potassium ion conversion. Where the mechanism of the CoTe_2_ phase transition reaction can be expressed as:

$$2{CoTe}_{2}+7K^{+}7e^{-}\to 2Co+K_{5}{Te}_{3}+K_{2}Te\;$$

$$2Co+K_{5}{Te}_{3}+K_{2}Te↔2CoTe+2Te+7K^{+}7e^{-}\;$$

The surface-driven reactions during rapid potassiumization/depotassiumization significantly promote the charge storage of CoTe_2_-C in potassium ion cells, leading to excellent rate performance. And the high contribution of the capacitance-controlled behavior of CoTe_2_-C indicates its good multiplicative performance. At a current density of 0.5 A g^−1^, the CoTe_2_-C composite has a 100th cycle discharge capacity of 189.5 mAh g^−1^ (Chen et al., 2019). In addition, Kim et al. (Fu and Lee, 2019) investigated a CuCo LDHs-coated CuCoTe honeycomb nanosheet as anode for hybrid SCs. They demonstrated the material’s excellent electrochemical performance and high stability (Chen et al., 2019).

### 3.3 Electrocatalysis

Depicted as 2D materials, tellurides have also been recently described as electrocatalytically active materials with low cost and strong catalytic activity (Luxa et al., 2017; Han et al., 2020). Nath et al. used hydrothermal and electrodeposition methods to create 5 and 8 nm-thick Ni_3_Te_2_ films (De Silva et al., 2018). Voltammetric cycling and linear sweep voltammetry were used to examine the OER catalytic activity of the as-synthesized materials in the alkaline electrolytes. Their results showed that Ni_3_Te_2_ films have a high catalytic efficiency of 10 mA cm^−2^ with a notably low overpotential of 180 mV. The overpotential value was much lower than that needed for nanostructured Ni_3_Se_2_ (190 mV) (Xu et al., 2019) and Ni_3_S_2_ (260 mV) (Li et al., 2022). Nanodendritic MoTe_2_ was used as an electrocatalyst for hydrogen precipitation reaction (Zhou et al., 2017a). Nanodendrimers were created electrochemically on Mo-doped reduced polyimide/graphene oxide composite substrates. The deposition period increased the size of the nanodendrites. Their findings further showed that nanodendrimers have the potential to be good catalysts for hydrogen precipitation in neutral fluids. Ashiq et al. proposed a highly active Cu_7_Te_4_ nanowire synthesized through water oxidation as an electrocatalyst for water oxidation reaction (Zhang et al., 2022). Chu et al. demonstrated the ability of FeTe_2_ to function as an efficient and durable nitrogen reduction electrocatalyst, with an excellent combination of NH_3_ yield and Faraday efficiency (Yao et al., 2022). Metal telluride materials, such as Sb_2_Te_3_ (He et al., 2022), MoTe_2_[99], Ni_3_Te_2_-CoTe[100], and NiTe-HfTe_2_[101], have received extensive attention for their potential application in electrocatalysis.

### 3.4 Li-S batteries

Because of its low cost, environmental friendliness, and high energy density, Li-S batteries are appealing next-generation energy storage technologies. Meanwhile, owing to the intrinsic 2D structure of telluride, it offers an interesting function in Li-S batteries (Wang et al., 2019; Guo et al., 2021; Hassan et al., 2021).

**FIGURE 8:**
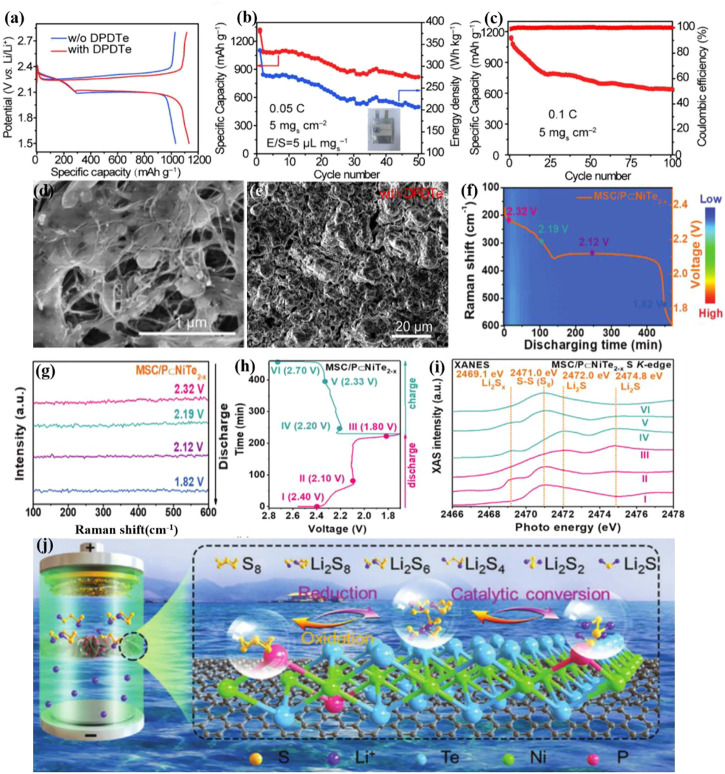
**(A–C)** Electrochemical performance of Li–S pouch cells with/without DPDTe additive, with high sulfur loading of 5 mg cm^−2^ and E/S ratio of 5.0 μL mg^−1^. **(D)** SEM images of the Li_2_S deposition with DPDTe and **(E)** the cycled Li anode with DPDTe additive at the 50th cycle[102]. Copyright 2022, Wiley-VCH. **(F,G)** *In situ* Raman contour plots and Raman spectra at 0.1 C with MSC/P⊂NiTe_2−x_; **(H,I)** Galvanostatic discharge/charge profiles and corresponding *ex situ* XANES of the S K-edge cathode with MSC/P⊂NiTe_2−x_ separator; **(J)** Adsorption-catalytic LiPS mechanism with MSC/P⊂NiTe_2−x_ in a Li–S configuration[103]. Copyright 2022, Wiley-VCH.

Xie et al. examined diphenyl ditelluride (DPDTe) as bifunctional electrolyte additive for high-efficiency sulfur cathodes and dendrite-free lithium anodes[102]. As shown in Figures 8A–E, the presence of DPDTe enables catalytic mediation by Te radicals, which is accountable for substantially enhancing LIPS redox dynamics and regulating Li_2_S accumulation. DPDTe can also combine with metallic lithium to generate a uniform, dense, and stable organic–inorganic hybrid SEI to minimize the nucleation overpotential of lithium and promote uniform lithium deposition, thereby successfully limiting the formation of lithium dendrites. The addition of DPDTe can improve sulfur usage and lead to highly reversible lithium stripping/plating, further resulting in good rate capability (611.4 mAh g^−1^ at 5C) of Li-S batteries.

Zhan et al. explored a phosphorus-doped nickel Te electrocatalyst (P⊂NiTe_2-x_) grown on carbon-based (MSC) as a functional layer for high-performance Li-S battery separators (MSC/P⊂NiTe_2-x_)[103]. The increased electrochemical performance implies that the P doping of Te vacancies can enhance Li-S battery conductivity, boost adsorption, and decrease the redox energy barrier of Li-S batteries. MSC nanosheets enable NiTe_2_ nanoparticles to disperse and diffuse Li^+^. *Ex-situ* X-ray absorption spectroscopy and *in-situ* Raman spectroscopy both demonstrated the ability of MSC/P⊂NiTe_2-x_ to inhibit the shuttle effect and accelerate the redox conversion (Figures 8F–J). Compounding telluride materials in the electrode[91, 104], electrolyte, and diaphragm coatings has become one of the important strategies of breaking through the severe shuttle effect of Li-S batteries.

## 4 Summary and prospective

Metal tellurides have received extensive attention owing to their great application potential for high-performance electrode materials. In this study, the synthesis methods of tellurides and the research progress of their properties and application in the field of electrodes were reviewed. Three methods for preparing metal tellurides were discussed. Then, the latest progress in terms of the role of telluride in capacitors, anode materials, electrocatalysis, and Li-S batteries was presented. Despite significant progress in the study of 2D tellurides, researchers still face considerable opportunities and challenges.

The CVD approach is now being widely utilized to controllably prepare tellurides. It is feasible to produce 2D tellurides with customizable shape and good crystallinity on a large scale. However, for powder electrochemical materials, the mild chemical interaction between the transition metal and Te under vapor conditions, on the other hand, is a disadvantage of CVD synthesis. Furthermore, while tellurides exhibit remarkable performance, they are difficult to precisely control properties such as pore structure and distribution in the preparation of electrode materials, which greatly affects the volume change of electrode materials during cycling, making it more difficult to improve the capacity, stability and extended cycle life of capacitors and batteries. Future studies should concentrate on: 1) investigating more approaches for the controlled synthesis of tellurides, not only for 2D single-crystal; and 2) designing more composites or building heterostructures to facilitate the electrochemical performances telluride-based electrodes, because like other sulfide generics, tellurides also suffer from extreme volume fluctuations, which result in poor cycling performance.
